# Supplementary material for: celsr1a is essential for tissue homeostasis and onset of aging phenotypes in the zebrafish
Source: eLife. 2020 Jan 27;9:e50523. doi: 10.7554/eLife.50523 (PMC7010407; doi:10.7554/eLife.50523)
Supplement: Supplementary file 1. — (A) Defined diets for zebrafish dietary restriction. (B) Primers used in the study. [file elife-50523-supp1.docx]

**Supplementary File 1**

**Supplementary File 1A: Defined diets for zebrafish dietary restriction.**

|  | **Control Diet (CD)** | | **25% Caloric Reduction (CR)** | | **50% Caloric Restriction** | |
| --- | --- | --- | --- | --- | --- | --- |
| **Component** | **% total** | **kcal** | **% total** | **kcal** | **% total** | **kcal** |
| Protein | 49.5 | 181.9 | 37 | 136 | 19.7 | 72.392 |
| Carbohydrate | 26.5 | 95.4 | 13 | 46.8 | 5.3 | 19.08 |
| Lipid | 12 | 94.25 | 12 | 94.25 | 12 | 94.25 |
| Fiber | 3 | - | 29 | 0 | 54 | 0 |
| Vitamins/Minerals | 9 | - | 9 | n/a | 9 | n/a |
| total |  | 371.55 |  | 277.05 (75%) |  | 185.72 (50%) |

**Recipe adjustments**

| **Ingredient** | **CD** | **25%CR** | **50%CR** |
| --- | --- | --- | --- |
| Wheat Gluten (g) | 15 | 11 | 6 |
| Casein (g) | 30.5 | 23 | 12 |
| Egg Whites (g) | 4 | 3 | 1.7 |
| Cellulose (g) | 3 | 29 | 54 |
| Starch (g) | 26.5 | 13 | 5.3 |
| Soybean Oil (g) | 7 | 7 | 7 |
| Ultralec lecithin (g) | 5 | 5 | 5 |
| Vitamin Mix (g) | 4 | 4 | 4 |
| Mineral Mix (g) | 4 | 4 | 4 |
| Stay C (g) | 1 | 1 | 1 |
| Total Weight (g) | 100 | 100 | 100 |
| Total Kcal (without vitamins & minerals) | 371.55 | 279.05 | 186.55 |

**Caloric content of ingredients**

(derived from indicated company websites)

| **Ingredient** | **Content** |
| --- | --- |
| Wheat Gluten (Dyets Inc. #402100) | 3.56 kcal/g |
| Casein (Dyets Inc. #400627) | 3.72 kcal/g |
| Egg Whites (Dyets Inc. #401600) | 3.76 kcal/g |
| Cellulose (Dyets Inc. #401850) | - |
| Vitamin Mix (Dyets Inc. #310069) | - |
| Mineral Mix (Dyets Inc. #210087) | - |
| Stay C* (Vitamin C-3, Argent Chemical Laboratories Inc.) | - |
| Starch (Baka-Snak Food Starch-Modified, National Starch Food Innovation) | 3.6 kcal/g |
| Lecithin (ADM, Ultralec without added tocopherol) | 6.25 kcal/g |
| Water (Milli-Q) | - |
| Tocopherol stripped soybean oil (Dyets Inc. #404365) | ~9.00 kcal/g |
| Vitamin E (Novatol 6-92, Archer Daniels Midland) | - |
|  |  |
| Total kcal per 100 g batch of control diet | 371.55 |
| Kcal/g of diet (not counting vit and mineral mixes) | 4.22 |

**Supplementary File 1B: Primers used in the study.**

| primer name | Sequence (5’-3’) | purpose |
| --- | --- | --- |
| sox2-Fw | GCTCTGCACATGAAGGAACA | *in situ* hybridization |
| sox2-Rv | TTTCCCTCCCCAAAAGAAGT |  |
| olfm4-Fw | GAGGTGATTCGCCATGAGTT | *in situ* hybridization |
| olfm4-Rv | AGCACCAAGACACTGCACAC |  |
| celsr1a-qPCR-F | CAACCTACCACCTCCTTTCTTC | qRT-PCR |
| celsr1a-qPCR-R | CAGCCTTCACTAGGTTCTCATT |  |
| celsr1b-qPCR-Fw2 | GACGGGTGTAATGTACCTGATG | qRT-PCR |
| celsr1b-qPCR-Rv2 | CACAGTCCCTGCCGAAATAA |  |
| celsr2-Fw | CATTGCGTTTGTGGTGTCTATG | qRT-PCR |
| celsr2-Rv | TCTCTTCTCGCAGCTCTTCT |  |
| celsr3-Fw | CCACTCAGAAGGAGATCAAGAAG | qRT-PCR |
| celsr3-Rv | CATCAGACCAAACAGCCAAAC |  |
| sirt1-F | GTCCAATCAGCAAACGACTCGGAG | qRT-PCR |
| sirt1-R | TCTTCATGCTGGAAAGATCCGTCG |  |
| sirt6-F3 | GAGGACAGGACACCTCAAATAC | qRT-PCR |
| sirt6-R3 | TGCCACACTTCTCACATTCT |  |
| cdnk1a/p21-F | AGCTGAAGCGCAAACAGA | qRT-PCR |
| cdnk1a/p21-R | GTAGATGCAGGTCAAGAGTTTATCT |  |
| pax7a-qPCR-Fw  pax7a-qPCR-Rv | GACACACTACCCTGACATCTAC  CCTTGCTCTTCTGTTGCTAAAC | qRT-PCR |
| deltaNp63-Fw  deltaNp63-Rv | GAGACCAATGCTCCCTCA  GGCTGGTGGATGTGGAG | qRT-PCR |
| cldnb-qPCR-Fw  cldnb-qPCR-Rv | ACAGATGCAGTGTAAGGTCTAC  ATTCCCATGACTCCGATCAC | qRT-PCR |
